# Risk factors and postnatal biomarkers for acute placental inflammatory lesions and intrauterine infections in preterm infants

**DOI:** 10.1007/s00431-022-04545-1

**Published:** 2022-07-14

**Authors:** Die Liu, Jing Liu, Fang Ye, Yunchao Su, Jiaoying Cheng, Qi Zhang

**Affiliations:** 1 Department of Pediatrics, China-Japan Friendship Hospital, Beijing, China; 2 Department of Pathology, China-Japan Friendship Hospital, Beijing, China; 3 Department of Obstetrics and Gynecology, China-Japan Friendship Hospital, Beijing, China

**Keywords:** Preterm infant, Placental inflammatory lesions, Intrauterine infection, Sialic acid

## Abstract

The purpose of this study is to explore risk factors of acute placental inflammatory lesions and the potential postnatal serum biomarkers for predicting the severity of intrauterine infection in preterm infants. We performed a retrospective analysis of premature infants with or without acute placental inflammatory lesions and their mothers by chart review for clinical data and placental histopathology. The preterm infants with acute placental inflammatory lesions had a higher rate of premature rupture of membranes (PROM), a longer duration of PROM, and a higher level of serum sialic acid (SIA) than those of the non-inflammation group (all *p* < 0.001). According to the different inflammatory histological structures, preterm infants with funisitis had a dominant longer duration of PROM than others (*p* < 0.05), and their gestational age was youngest among all the infants (*p* < 0.05). Furthermore, they had the highest content of serum SIA above other groups. The preterm infants in the acute histological chorioamnionitis group showed a similar trend of clinical manifestation and laboratory parameters with the funisitis group. Moreover, the closer the placental lesions were to the fetus, the lower the gestational age of preterm infants was, and the higher the serum SIA content was.

*Conclusion*: We utilized a simple and precise anatomically category method of placental inflammatory histopathology for pediatricians to distinguish the extent of fetal inflammatory response for representing early-onset infectious diseases of preterm infants. SIA might be one of the potential early-stage serum biomarkers to reflect the severe intrauterine infections and could guide the postnatal anti-infection treatment.

**Table:** 

**What is Known:** • *Acute placental inflammatory lesion contributes to preterm birth and a series of complications in preterm infants.* • *C-reactive protein and interleukin-6 in neonatal blood can be used as biomarkers for potential early-onset sepsis, but they are influenced by the postnatal physiological changes of preterm infants.*	
**What is New:** • *The value of serum sialic acids of preterm infants within 1-hour afterbirth may be one of the rapid postnatal biomarkers for evaluating the severity of intra-amniotic infection.* • *The closer the placental lesions are to the fetus, the higher the content of serum sialic acid is.*

**What is Known:**

• *Acute placental inflammatory lesion contributes to preterm birth and a series of complications in preterm infants.*

• *C-reactive protein and interleukin-6 in neonatal blood can be used as biomarkers for potential early-onset sepsis, but they are influenced by the postnatal physiological changes of preterm infants.*

**What is New:**

• *The value of serum sialic acids of preterm infants within 1-hour afterbirth may be one of the rapid postnatal biomarkers for evaluating the severity of intra-amniotic infection.*

• *The closer the placental lesions are to the fetus, the higher the content of serum sialic acid is.*

## Introduction

Acute inflammatory lesions of the placenta, characterized by diffuse infiltration of neutrophils into the placental disc, chorioamniotic membranes, and umbilical cord, are generally considered intra-amniotic infections [1]. Regarding the crucial interactive role of the placenta between the mother and the developing fetus, the inflammatory response of the placenta is particularly complicated, and the histopathological changes of the placenta may directly lead to adverse pregnancy outcomes, especially the preterm labors [2]. A study including 7505 neonates showed the proportion of chorioamnionitis in preterm infants from 21 to 37 weeks of gestation was 18.7%, while that in full-term babies was only 3.9% [1]. However, acute inflammatory lesion of the placenta is not only the cause of preterm delivery but also one of the main factors contributing to a series of complications in preterm infants which may contribute to long-term sequelae or even chronic disease in adulthood [3–5].

Depending on different anatomical structures of the placenta and its affiliations, inflammatory lesions may give rise to various clinical significance [1]. Previous studies have shown that clinical chorioamnionitis or amniotic cavity infection was closely related to neonatal early-onset sepsis (EOS), especially in preterm infants [6, 7]. There was evidence showing benefits for classifying preterm infants by the placental histopathologic features [8]. Acute inflammatory lesion of the placenta is a wider concept than histological chorioamnionitis (HCA), though the definition of the acute inflammatory lesion was often replaced by HCA because several grading and staging systems were simultaneously applied to identify the severity of acute chorioamnionitis [9, 10]. But most classifying systems are not conducive to the practical application for both pathologists and clinicians [10].

In this article, we used acute subchorionitis, chorionitis, HCA, and funisitis to distinguish the different structural lesions of the placenta based on the anatomical regions from maternal to fetal part, to simplify the grading and staging system to describe the maternal and fetal inflammatory response, which might be helpful to find a more accurate correlation between the clinical outcomes of preterm babies and the perinatal characteristics of their mothers. Therefore, the present study aims to exert a retrospective analysis of clinical data in the perinatal period to explore risk factors of acute inflammatory lesions of the placenta from a histopathological perspective and to investigate potential serum biomarkers for predicting the severity of intra-amniotic infection in preterm infants.

## Methods

### Patient selection

Maternal and neonatal charts were reviewed retrospectively for clinical features, laboratory data, and placental pathological examinations for infants born at less than 37 weeks of gestation from January 1, 2018, to December 31, 2020, at China-Japan Friendship Hospital. Inclusion criteria were as follows: (1) infants were born with a gestational age of fewer than 37 weeks; (2) their placentas were taken pathological examinations; (3) the medical records of maternal application of glucocorticoids and antibiotics were accurate. The following exclusion criteria included the following: (1) neonates with congenital abnormalities, chromosome anomalies, or genetic and metabolic disorders; (2) family members decided to quit treatment within 24 h after infants’ birth causing incomplete clinical records during hospitalization; (3) family members refused the placental pathological examination.

### Placental pathology and group criteria

The placental pathological examination was performed in the mothers who gave preterm labor in our hospital. After delivery, the placenta disc, fetal membranes, and umbilical cord tissues were sent for pathology within 24 h. Specimens were collected by pathologists, and gross and microscopic observations were conducted.

Diagnosis criteria of acute inflammatory lesions of the placenta complied with the Amsterdam Placental Workshop Group Consensus Statement [9]. Generally, the inflammatory lesions of the placenta were divided into acute inflammatory lesions, chronic inflammatory lesions, and mixed inflammatory lesions depending on the type of inflammatory cells. Also, there were non-inflammatory lesions of the placenta, such as small calcification and vascular malperfusion. In this study, we focused on acute inflammatory lesions and non-inflammatory lesions.

Considering the shortage of feasibility and effectiveness of the existing category system of acute placental inflammatory lesions, we defined neutrophils infiltration according to the placental histological structure from maternal to fetal side and divided the subjects into four subgroups: acute subchorionitis (including deciduitis), chorionitis, HCA, and funisitis. In addition, due to the inadequate number of infiltrating neutrophils to be given a concrete diagnosis, we nominated this situation as “suspected inflammation” which was the fifth subgroup in the study. The clinical data of mothers and premature infants in 5 subgroups were analyzed.

### Clinical data collection

Clinical data and pathological data were collected from obstetrical and neonatal medical records, including (1) general information: (a) maternal chart—maternal age, parity, amniotic fluid situation, pregnancy complications, delivery methods, antenatal antibiotics, and dexamethasone usage. The indications for prophylactic antibiotics treatment include suspected clinical chorioamnionitis, prelabor rupture of membranes, positive streptococcus agalactiae culture of vagina secretion, and prolonging vaginal bleeding because of placenta previa. (b) infant chart—gestational age, gender, birth weight, Apgar score at 1 min, fetal distress, and premature rupture of membranes (PROM). (2) Neonatal hematological indices were assessed in medical laboratory of China-Japan-Friendship Hospital as routine blood tests within 1 h after birth. The parameters included the total number and classification of white blood cells (WBCs), platelet (PLT) count, C-reactive protein (CRP), phospholipids, serum sialic acids (SIAs), superoxide dismutase (SOD), monoamine oxidase (MAO), and α-l-fucosidase (AFU). (3) Clinical complications and outcomes of infants during hospitalization—usage of mechanical ventilation, intrauterine infectious complications (intrauterine pneumonia, neonatal EOS, purulent meningitis, urinary tract infection, etc.), neonatal respiratory distress syndrome (NRDS), patent ductus arteriosus (PDA), necrotizing enterocolitis (NEC), bronchopulmonary dysplasia (BPD), and hypoxic-ischemic encephalopathy (HIE). The diagnostic criteria for maternal diseases referred to Williams Obstetrics (25th Edition) [11], while that of neonates’ referred to the fourth edition of Practical Neonatology [12].

### Statistical analysis

Statistical analysis was performed using SPSS 23.0 software. Quantitative data were compared with *t* test and one-way ANOVA followed by Dunnet’s tests (parametric) or with Kruskal–Wallis rank test followed by Dunn’s multiple comparison tests (nonparametric). Normally distributed data were described by means ± standard deviation (means ± SD), while non-normally distributed data were described by median (interquartile). The qualitative data were expressed as proportion (%), and comparisons between groups were done with chi-square (*χ*^2^) test or Fisher’s exact test. Logistic regression analysis was used to analyze the correlation between the risk factors/biomarkers and acute inflammatory lesions of the placenta. A *p*-value of < 0.05 was considered statistically significant.

## Results

### Correlation between acute inflammatory lesions of the placenta and the perinatal clinical characteristics of mothers and offspring

**Fig. 1:**
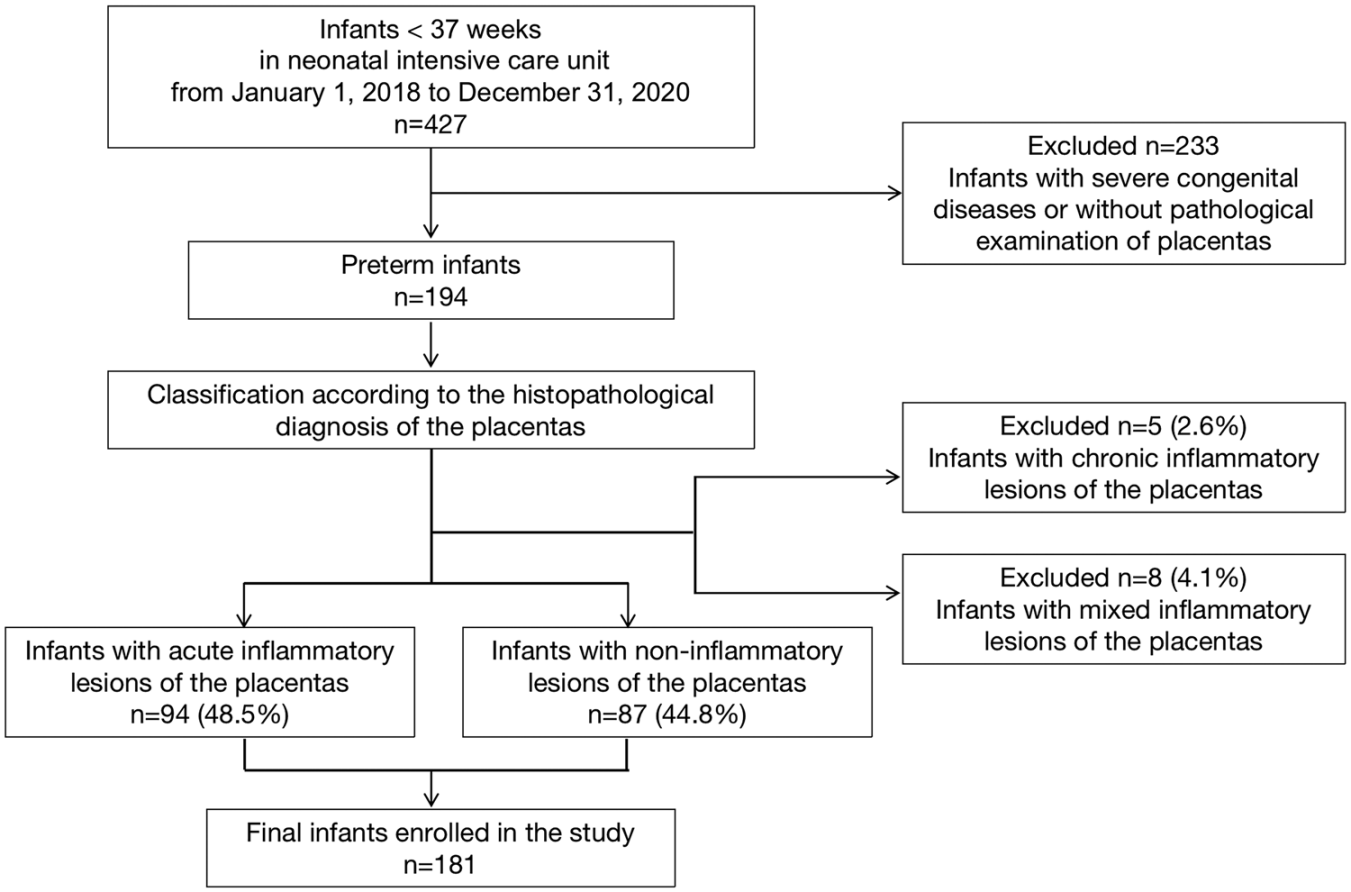
Flow chart of infant enrollment criteria in the study. n, number of infants

A total of 427 premature infants were admitted and treated in the neonatal intensive care unit during the study period. According to the inclusion and exclusion criteria, 194 preterm infants conformed to the criteria. Based on the placental pathology, 94 cases were diagnosed as acute placental inflammatory lesions, while 87 cases belonged to the non-inflammatory lesion group, and the remaining 13 infants had chronic or mixed inflammatory lesions. Finally, a total of 181 cases were included in this study (Fig. 1).

Compared between the acute inflammatory lesion group and the non-inflammatory lesion group, there was no significant difference in premature infant’s gender, gestational age, birth weight, the incidence of fetal distress, and amniotic fluid contamination (all *p* > 0.05). The occurrence and duration of PROM in the acute inflammatory lesion group were dramatically increased compared with those in the non-inflammatory lesion group (both *p* < 0.001). In concordance with a higher proportion of complete dexamethasone treatment before delivery to accelerate fetal lung maturity in the acute inflammatory lesion group (*p* < 0.05), the number of cases receiving preventive antibiotics treatment in this group was nearly four times that in the non-inflammatory lesion group (*p* < 0.05). The maternal childbearing age and parity were not related to the presence of acute inflammatory lesions in the placenta (both *p* > 0.05). In the non-inflammatory lesion group, the incidence of gestational hypertension and that of intrahepatic cholestasis of pregnancy were obviously higher than these of the acute inflammatory lesion group, and the differences were statistically significant (both *p* < 0.05). There was no significant difference in the incidence of gestational diabetes mellitus, placenta previa, placental abruption, hypothyroidism, prenatal fever, and HELLP syndrome between the two groups (all *p* > 0.05). In the respect of delivery mode, the mothers in the acute inflammatory lesion group were mostly delivered vaginally (*p* < 0.001) (Table 1).

**Table 1:** Comparison of clinical characteristics of premature infants and their mothers in acute placental inflammatory lesion group and non-inflammatory lesion group

	Acute inflammatory lesion group (*N* = 94)	Non-inflammatory lesion group (*N* = 87)	Statistical value	*p* value
General information of preterm infants				
Male	63(67.0%)	45(51.7%)	4.393	0.036
Gestational age (weeks)	34.25 ± 2.17	34.69 ± 1.92	− 1.235	0.217
Birth weight (g)	2239.20 ± 569.08	2302.64 ± 552.73	− 0.795	0.427
Delivery information				
Vaginal delivery	46(48.9%)	15(17.2%)	20.314	< 0.001
Fetal stress	19(20.2%)	14(16.1%)	0.515	0.473
PROM	52(55.3%)	24(27.6%)	14.266	< 0.001
Duration of PROM (hours)	8.5(0, 54)	0 (0, 2)	− 4.372	< 0.001
Amniotic fluid contamination	4(4.3%)	6(6.9%)	0.604	0.437
Clinical data of mothers				
Age (years)	32.27 ± 3.58	32.97 ± 4.36	− 1.735	0.083
Parity 1	62(66.0%)	59(67.8%)	0.1	0.95
Administration of antibiotics within 12 h before labor	46(48.9%)	11(12.6%)	27.584	< 0.001
Complete Dex therapy	58(61.7%)	41(47.1%)	3.874	0.049
Pregnancy complications				
GDM	31(33%)	23(26.4%)	0.924	0.336
Gestational hypertension	9(9.6%)	20(23.0%)	6.042	0.014
Placenta previa	9(9.6%)	15(17.2%)	2.309	0.129
Placental abruption	3(3.2%)	6(6.9%)	1.313	0.252
Hypothyroidism	11(11.7%)	15(17.2%)	1.127	0.288
HELLP syndrome	2(2.1%)	1(1.1%)	0.265	0.607
ICP	0(0%)	5(5.7%)	5.556	0.018
Prenatal fever	5(5.3%)	1(1.1%)	2.451	0.117

Quantitative data: normally distributed data are represented by mean ± SD; non-normally distributed data are described by median (interquartile); and the statistical value is *t*. Qualitative data: expressed as *N* (%), the statistical value is *χ*.^2^. *Dex* dexamethasone, *PROM* premature rupture of membranes, *GDM* gestational diabetes mellitus, *ICP* intrahepatic cholestasis of pregnancy

### Association between acute inflammatory lesions of the placenta and early laboratory examinations and complications in preterm infants

The preterm infants with the acute placental inflammatory lesions had a 14.5% higher concentration of serum SIA within 1 h after birth than that of the non-inflammation group (*p* < 0.001). But the WBC count and the counts of WBC category, PLT count, CRP, SOD, MAO, AFU, and phospholipids in serum showed no statistical difference between the two groups (all *p* > 0.05). The results exhibited no significant difference in Apgar score at 1 min, the application of mechanical ventilation, and the prevalence of intrauterine infectious complications, NRDS, PDA, BPD, NEC, and HIE of the premature babies between the two groups (all *p* > 0.05) (Table 2).

**Table 2:** Early laboratory examinations, clinical outcomes, and complications of preterm infants compared between acute placental inflammatory lesion group and non-inflammatory lesion group

	Acute placental inflammation (*n* = 94)	No inflammation (*n* = 87)	Statistical value	*p* value
Apgar score at 1 min ≤ 7	7	8	0.182	0.670
Hematological tests within 1 h after birth				
WBC count (× 10^9^/L)	12.83 ± 5.3	12.49 ± 5.43	− 0.701	0.483
Neutrophils count (× 10^9^/L)	7.24 ± 3.45	7.0 ± 3.85	− 0.864	0.388
Lymphocytes count (× 10^9^/L)	3.56 ± 1.25	3.83 ± 1.61	− 0.611	0.541
PLT count (× 10^9^/L)	250 ± 67	246 ± 64	− 0.647	0.517
Abnormality of CRP	11 (11.7%)	5 (5.7%)	1.988	0.159
SOD (U/ml)	210.3 ± 56.8	197.5 ± 43.6	− 1.556	0.12
MAO (U/L)	6.92 ± 3.63	6.49 ± 3.75	− 1.261	0.207
Phospholipids (mmol/L)	1.51 ± 0.24	1.48 ± 0.32	− 1.354	0.176
AFU (U/L)	27.20 ± 9.65	25.86 ± 7.96	− 1.106	0.269
SIA (mg/dL)	24.63 ± 5.71	21.52 ± 3.81	− 3.558	< 0.001
Invasive mechanical ventilation	15 (16.0%)	12 (13.8%)	0.167	0.693
Days of ventilation	0 (0, 0)	0 (0, 0)	− 0.521	0.602
Noninvasive mechanical ventilation	41 (43.6%)	31 (35.6%)	1.203	0.273
Days of ventilation	0 (0, 5.37)	0 (0, 3)	− 1.299	0.194
Intrauterine infectious complications	84 (89.4%)	69 (79.3%)	3.491	0.062
Intrauterine pneumonia	81 (86.2%)	68 (78.2%)	1.991	0.158
Early-onset sepsis	10 (10.6%)	7 (8.0%)	0.357	0.550
Purulent meningitis	2 (2.1%)	0 (0%)	1.872	0.171
Urinary tract infection	1 (1.0%)	0 (0%)	0.931	0.335
Cutaneous infection	2 (2.1%)	0 (0%)	1.872	0.171
NRDS	23 (24.5%)	19 (21.8%)	0.175	0.675
PDA	8 (8.5%)	4 (4.6%)	1.118	0.29
BPD	2 (2.1%)	1 (1.1%)	0.265	0.607
NEC	5 (5.3%)	1 (1.1%)	2.451	0.117
HIE	5 (5.3%)	3 (3.4%)	0.156	0.583

Quantitative data: normally distributed data are represented by mean ± SD, non-normally distributed data are described by median (interquartile), and the statistical value is *t*. Qualitative data: expressed as *N* (%), the statistical value is *χ*.^2^. *WBC* white blood cell, *PLT* platelet, *CRP* C-reactive protein, *SOD* superoxide dismutase, *MAO* monoamine oxidase, *AFU* α-l-fucosidase, *SIA* sialic acids, *NRDS* neonatal respiratory distress syndrome, *PDA* patent ductus arteriosus, *BPD* bronchopulmonary dysplasia, *NEC* necrotizing enterocolitis, *HIE* hypoxic-ischemic encephalopathy

### Exploration of risk factors and potential serum biomarkers of acute inflammatory lesions of the placenta

We examined the possible high-risk factors of acute placental inflammatory lesions in preterm infants by logistic regression analysis and it showed that the duration of PROM, the administration of antibiotics before delivery, and the serum SIA of infants were all the risk factors of acute placental inflammatory lesions. The choices of delivery methods and antibiotic treatments were both medical interventions that obstetricians considered it necessary to adopt, so the statistical results of the two factors could not indicate the predictive risk efficiency for acute placental inflammatory lesions. Therefore, the duration of PROM might be one of the independent risk factors for acute placental inflammatory lesions, and meanwhile, the higher the concentration of serum SIA in infants was, the greater the possibility of acute inflammatory lesions occurring in the placenta was (Table 3).

**Table 3:** Analysis of risk factors for acute inflammatory lesions of the placenta

	Odds ratio	95% CI	*p* value
PROM	3.250	1.746 ~ 6.051	< 0.001
Cesarean section vs vaginal delivery	0.217	0.109 ~ 0.432	< 0.001
Administration of antibiotics within 12 h before labor	6.621	3.126 ~ 14.025	< 0.001
Serum SIA	1.158	1.074 ~ 1.249	< 0.001

*CI* confidence interval, *PROM* premature rupture of membranes, *SIA* sialic acids

### Comparison of early clinical indicators and infectious complications in preterm infants among anatomical subgroups of acute placental inflammatory lesions

According to the different histological structures and degrees of neutrophil infiltration, the acute inflammation group was divided into 5 subgroups: acute subchorionitis group, acute chorionitis group, acute HCA group, acute funisitis group, and the suspected inflammation group (the degree of neutrophils infiltration was not sufficient to give a diagnosis of acute inflammation). The classification rule of the previous 4 subgroups conformed to the anatomical continuity from maternal to fetal side of the placenta as well as to maternal to fetal response to infection. Next, we observed that there were still significant differences in the risk factors (gestational age, the duration of PROM, antibiotic administration rate, and serum SIA of premature infants) among the 5 subgroups and the non-inflammatory lesion group (all *p* < 0.05). Furthermore, all of the infants whose placentas were diagnosed pathologically with HCA (12 cases) and funisitis (7 cases) suffered from intrauterine infectious diseases though there was no significance statistically in the occurrence of infectious complications among the 6 groups (Table 4).

**Table 4:** Potential risk factors and biomarkers for various histological inflammatory lesions of the placenta

	*N* (%)	Gestational age^a^ (weeks)	Duration of PROM^b^ (hours)	Serum SIA^a^ (mg/dL)	Administration of antibiotics before labor	Intrauterine infectious complication
Non-inflammation	87 (48.1)	34.69 ± 1.92	0 (0,2)	21.52 ± 3.81	11 (12.6%)	69 (79.3%)
Suspected inflammation	12 (6.6)	34.95 ± 1.72	10 (0,25.25)	22.33 ± 2.81	4 (33.3%)	10 (83.3%)
Subchorionitis	4 (2.2)	35.36 ± 1.39	12 (1,41)	23.25 ± 1.71	1 (25.0%)	3 (75%)
Chorionitis	59 (32.5)	34.56 ± 2.08	0 (0,48)	23.58 ± 4.65	30 (50.8%)	52 (88.1%)
HCA	12 (6.6)	33.18 ± 2.47	14.5 (0,59.5)	27.1 ± 7.72	4 (33.3%)	12 (100%)
Funisitis	7 (3.9)	31.65 ± 0.68	96 (60,168)	34 ± 5.86	7 (100%)	7 (100%)
Statistical value	–	18.397	27.583	27.34	39.915	6.167
*p* value	–	0.002	< 0.001	< 0.001	< 0.001	0.29

*PROM* premature rupture of membranes, *SIA* sialic acids, *HCA* histological chorioamnionitis

^a^Described as mean ± SD

^b^described as median (interquartile). Data were analyzed by Kruskal–Wallis test

**Fig. 2:**
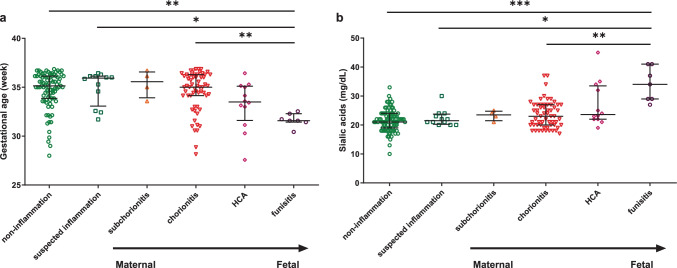
Comparison of gestational age **a** and serum sialic acids content **b** in different subgroups of placental pathology in premature infants. HCA, histological chorioamnionitis. *p < 0.05, **p < 0.01, ***p < 0.001

Premature infants with funisitis had a dominant longer duration of PROM and a higher rate of antibiotics usage before birth than these of other babies (all *p* < 0.05). The infants in the funisitis group were 1.53 to 3.71 weeks younger than these preterm neonates with other placental lesions, and they had the highest content of serum SIA above other groups. Likewise, these parameters in the acute HCA group displayed a similar trend with the funisitis group, although the magnitude of the change was smaller than that of the funisitis group. Moreover, the closer the placental lesions were to the fetus, the lower the gestational age of preterm infants was, and the higher the serum SIA level was (Fig. 2).

## Discussion

Placental inflammatory lesion is the immune response of mother and fetus to intrauterine infection [13]. The general definition of HCA refers to the infiltration of neutrophils in the placenta, fetal membranes, and umbilical cord which would lead to adverse pregnancy outcomes, such as premature delivery, PROM, and neonatal EOS [13, 14]. The premature birth rate with HCA was about twice that without HCA [15]. The mechanism might be that when HCA occurs, bacterial endotoxins or exotoxins are recognized by Toll-like receptors and stimulate the placental tissue to produce inflammatory cytokines which could induce prostaglandin release and directly initiate uterine contractions [16] or trigger T cell proliferation in response to maternal antigens through pro-inflammatory reactions via interferon-γ and tumor necrosis factor-α pathways [17]. Previous studies indicated that HCA or amniotic cavity infection and neonatal EOS were closely connected [6, 7]. Moreover, Apgar score, endotracheal intubation rate, mechanical ventilation rate, pulmonary surfactant application, severe PDA rate, and the incidence of BPD in very low birth weight infants were related to HCA [18]. Maternal amnionitis led to the low gestational age and low birth weight of preterm infants, and it was an independent risk factor for intraventricular hemorrhage [19]. Therefore, it is important to unmask the predictive biomarkers of intrauterine infection in the early stage to reduce the complications of preterm infants by an adequate postnatal follow-up.

PROM and intrauterine infection are reciprocal causation [13]: PROM could motivate pathogen to retrograde causing intrauterine infection, while inflammatory factors released during intrauterine infection could trigger PROM. Preterm PROM was responsible for about one-third of the preterm delivery rate [20], and with the prolongation of premature rupture of membranes, especially those with premature rupture longer than 24 h, the probability of EOS would increase by 2–10 times [21]. In this study, it was found that PROM was one of the independent risk factors for acute inflammatory lesions of the placenta. The vaginal delivery rate and PROM rate were higher in the group of acute placental inflammatory lesions, and the duration of PROM in this group was obviously longer than that in the non-inflammation group. Analysis of anatomical subgrouping revealed that the longer the duration of PROM lasts, the closer the neutrophils infiltration was to the fetal side. However, in the present research, there was no statistically significant association between the acute placental inflammatory lesions and the infectious complications of premature infants. This result might be explained by the fact that the antepartal intervention of antibiotics could influence the prevalence of infectious diseases of preterm babies. The duration of PROM is often used to predict the possibility of intrauterine infection, so as to apply timely intervention and to minimize the occurrence of infection-related complications in preterm infants. Since there is a lack of tools to accurately predict intrauterine infection before delivery, the combination of the time of PROM, the contamination of amniotic fluid, and the clinical manifestations of pregnant women to anticipate intrauterine infection is still one of the most effective methods as the guidance of applying antibiotics in advance to diminish the intrauterine infectious complications of preterm infants. However, there was only 5.3% of maternal fever occurring in the acute inflammation group, and 1.1% of pregnant women in the non-inflammation group getting prenatal fever which might be considered “isolated maternal fever” [13], suggesting that maternal fever is not a reliable marker of intrauterine infection. Although placental gross and histological examination should always stand as a gold standard in preterms, its turn-around-time is sometimes long. Therefore, neonatologists/pediatricians need an additional objective indicator to evaluate whether the newborns suffer intrauterine infections and whether the intrauterine infections have been effectively controlled.

We discovered that serum SIA in preterm infants might be one of the postnatal biomarkers of intrauterine infection. Interleukin (IL)-6 in amniotic fluid is specific as an antenatal biomarker for intrauterine infection, but it is still controversial to use it in clinical practice not just because the way to acquire the sample is invasive [13]. Though the levels of CRP, procalcitonin, IL-6, and IL-8 in umbilical cord blood or neonatal blood could be used as postnatal biomarkers for potential EOS, getting a sufficient amount of blood samples is most difficult in clinical practice, and those biomarkers are influenced by the postnatal physiological changes [22]. Therefore, they are not practical postnatal biomarkers for intrauterine infections. In our study, we observed that the serum SIA level of preterm infants with acute placental inflammatory lesions was significantly higher than that of the non-inflammation group. But at the same time, the CRP displayed no variation between the two groups, suggesting that serum SIA might be one of the indicators with higher sensitivity to predict intrauterine inflammation in the early postnatal period.

SIA, also known as sialome, is a generic term for a family of nine carbon monosaccharides, which are derivatives of neuraminic acid. The main forms in vertebrates are N-acetylneuraminic acid (Neu5Ac), N-glycolylneuraminic acid (Neu5Gc), and deaminoneuraminic acid (Kdn) [23]. SIA isoforms establish SIA cycle in the body, including the participation in the sialylation modification of glycoproteins and glycolipids in cells’ surfaces for providing the recognition sites for the formation of glycocalyxes which can manipulate the cellular interaction of immune cells, nerve cells, and other cells [21, 23]. Previous studies showed that SIA played a predominant role in the biological process of breast cancer metastasis, atherosclerosis, pancreatic cancer, immune response of nervous system, atopic diseases, and muscle aging [24–29]. The normal value of serum SIA in full-term infants is (27.1 ± 1.7) mg/dL for male newborns and (27.3 ± 1.7) mg/dL for females, but there is no sex-linked difference [30]. A single-center study has shown that the serum SIA level of preterm infants was lower than that of full-term infants (26.6 ± 7.5 vs 29.8 ± 9.3) mg/dL, suggesting that serum SIA concentration might be positively correlated with gestational age [31]. SIA was also engaged in the fetal lung maturation since the elevated expression of SIA in fetal lung indicated the better differentiation of bronchoalveolars [32].

After the further analysis of histopathological subgroups, it was interesting to discover that although the gestational age of preterm infants with funisitis was significantly lower than that of other groups, the content of serum SIA, which should gradually drop down accompanied by the decrease of gestational age, was about 58.0% higher than that of non-inflammatory lesion group. Similarly, HCA group showed an upregulating serum SIA level (about 26.0% higher than that of non-inflammatory lesion group) though the difference did not reach statistical significance. The infectious complication ratios of newborns in the funisitis group and the HCA group were both 100%, implying that when neutrophils infiltrated into the amniotic membrane, the serum SIA level of preterm infants increased substantially, representing a greater possibility of fetal inflammatory response and high prevalence of infectious complications in premature babies. This result further confirmed that the serum SIA content might be one of the potential early-stage biomarkers to reflect the intrauterine fetal inflammatory response, whose sensitivity within 1 h after birth was prior to that of CRP, and it might play a guiding role in the early postnatal anti-infection treatments. In addition, an animal experiment revealed the SIA biological function on the nervous system. After the newborn rats were exposed to lipopolysaccharide, the neuraminidase (Neu) 1 and Neu4 were upregulated in the brain tissue through the interaction of sialyltransferase and Neu, resulting in the continuous downregulation of neuronal glycoprotein sialylation, which might be one of the mechanisms of perinatal inflammatory stimulation leading to neonatal neuropathological sequelae [33]. Thereby, it showed that the increased level of serum SIA in premature infants with acute placental inflammatory lesions probably reflected a decreased sialylation modification of cells in vivo, especially attributing to the change of glycosylation degree on the cell membrane of the immune system and nervous system, which could influence a long-term prognosis of nervous system development of preterm infants. However, we still need further follow-up studies to evaluate the association of free SIA and nervous complications of preterm newborns.

## Limitations

The present study had some limitations. First, it is a single-center retrospective study with a small case number, so each subgroup by the anatomical categories of placental lesions shared less case number, which could affect a real clinical significance. Second, also due to the small sample number, it is impossible to calculate the cutoff value of serum SIA between the funisitis group and the non-inflammatory group. Third, as described above, the investigation of SIA’s potential biological function on the nervous system needs, at least, a follow-up evaluation of infants’ neurodevelopment.

## Conclusion

In summary, our study provides a simple and precise biochemical and clinical method based on placental inflammatory histopathology to distinguish the degrees of fetal pre- and post-natal inflammatory response, which might better guide a follow-up in preterm babies. The present study sheds light on one potential early-stage biomarker, SIA, in premature infants for predicting severe intrauterine infections to direct postnatal anti-infection treatment as soon as possible. However, it still remains further exploration of the mechanism in (1) the association of free SIA serum level and sialylation modification in biological functional regulation of preterm infants, and (2) the potential role of sialylation of cells in the infant’s long-term sequelae which could provide more concrete evidence for the predicting value of SIA in clinical practice.

## Data Availability

Not applicable.

## Abbreviations

AFU: α-L-Fucosidase
BPD: Bronchopulmonary dysplasia
CRP: C-Reactive protein
EOS: Early-onset sepsis
GDM: Gestational diabetes mellitus
HCA: Histological chorioamnionitis
HIE: Hypoxic-ischemic encephalopathy
ICP: Intrahepatic cholestasis of pregnancy
IL: Interleukin
Kdn: Deaminoneuraminic acid
MAO: Monoamine oxidase
NEC: Necrotizing enterocolitis
Neu: Neuraminidase
Neu5Ac: N-Acetylneuraminic acid
Neu5Gc: N-Glycolylneuraminic acid
NRDS: Neonatal respiratory distress syndrome
PDA: Patent ductus arteriosus
PLT: Platelet
PROM: Premature rupture of membranes
SIA: Serum sialic acids
SOD: Superoxide dismutase
WBCs: White blood cells
